# Current treatment and outcomes of traumatic sternal fractures—a systematic review

**DOI:** 10.1007/s00264-018-3945-4

**Published:** 2018-04-26

**Authors:** Dorine S. Klei, Mirjam B. de Jong, F. Cumhur Öner, Luke P. H. Leenen, Karlijn J. P. van Wessem

**Affiliations:** 1 Department of Trauma Surgery, University Medical Centre Utrecht, Suite G04.232, Heidelberglaan 100, 3584 CX Utrecht, The Netherlands; 2 Department of Orthopaedic Surgery, University Medical Centre Utrecht, Utrecht, The Netherlands

**Keywords:** Traumatic sternal fractures, Treatment, Outcomes, Systematic review

## Abstract

**Purpose:**

Traumatic sternal fractures are rare injuries. The most common mechanism of injury is direct blunt trauma to the anterior chest wall. Most (> 95%) sternal fractures are treated conservatively. Surgical fixation is indicated in case of fracture instability, displacement or non-union. However, limited research has been performed on treatment outcomes. This study aimed to provide an overview of the current treatment practices and outcomes of traumatic sternal fractures and dislocations.

**Methods:**

A systematic review of literature published from 1990 to June 2017 was conducted. Original studies on traumatic sternal fractures, reporting sternal healing or sternal stability were included. Studies on non-traumatic sternal fractures or not reporting sternal healing outcomes, as well as case reports (*n* = 1), were excluded.

**Results:**

Sixteen studies were included in this review, which reported treatment outcomes for 191 patients. Most included studies were case series of poor quality. All patients showed sternal healing and 98% reported pain relief. Treatment complications occurred in 2% of patients.

**Conclusions:**

Treatment of traumatic sternal fractures and dislocations is an underexposed topic. Although all patients in this review displayed sternal healing, results should be interpreted with caution since most included studies were of poor quality.

## Introduction

Sternal fractures are rare injuries with an incidence of less than 0.5% of all fractures and an estimated 3–8% in blunt trauma patients [1–4]. Traumatic sternal dislocations occur even less frequently [5]. The most common mechanism of injury is direct blunt trauma to the anterior chest caused by motor vehicle accidents [1, 6–8]. The incidence of sternal injury has increased since the introduction of seatbelt legislation [3, 9, 10]. Additionally, sternal injuries are frequently caused by falls from height or indirect trauma due to spinal flexion-compression injury [1, 2, 5, 6, 11]. Traumatic sternal fractures are mostly transverse sternal body fractures, while manubrial and xiphoid fractures occur less frequently [3, 8, 10]. Two types of sternal dislocations are distinguished: the sternal body is dislocated either posteriorly (type 1) or anteriorly (type 2) to the manubrium [2, 5, 7, 12].

An isolated sternal fracture is seen as a relatively benign injury [2, 3, 6]. Morbidity and mortality of sternal fractures are mostly determined by concomitant injuries of internal thoracic organs and mortality rates range from 4 to 45% [2, 3, 10]. Frequently encountered associated thoracic injuries include vertebral fractures (particularly of the cervical and thoracic spine), rib fractures, clavicular fractures, scapular fractures, pulmonary contusion, haemopneumothorax, cardiac and mediastinal injury, and aortic dissection [2, 9, 10, 13]. Other commonly associated injuries include brain injury and abdominal injury [3, 9]. Concomitant injuries and severe chest pain could lead to respiratory insufficiency, organ failure, and ultimately mortality [1, 2].

The majority of sternal fractures (> 95%) is treated conservatively [1, 3, 10, 14]. Conservative treatment options consist of analgesia, corset fixation, rest, and passive reduction of displacement if necessary [1, 15]. Adequate analgesia is of vital importance to prevent pulmonary complications caused by respiratory insufficiency as a consequence of painful respiration [15, 16]. However, in case of unstable fractures, thoracic wall instability, fracture displacement or persistent dislocation, sternal deformity, respiratory insufficiency, severe pain, and fracture non-union, surgical fixation could be performed [1, 2, 4, 5, 7, 10, 17]. Several fixation methods have been described in literature, of which wiring and plating are most regularly used [2, 5, 6, 11, 17]. Biomechanically, surgical plating provides more stability and a better restoration of anterior chest wall function than wiring, and recent evidence suggests that plating results in improved bone healing and decreased complications and non-union [1, 2, 6, 7, 17, 18].

Few studies have been published about the (long-term) treatment outcomes of either conservative or surgical treatment of traumatic sternal fractures and dislocations [6, 7]. No randomised controlled trials (RCTs) have been conducted on this topic. To our knowledge, only one systematic review has been conducted by Harston and Roberts in 2011 which focussed on surgical fixation of sternal fractures [4]. However, no systematic review has compared conservative and operative treatment of sternal fractures or dislocations. The aim of this study was to conduct a systematic literature review to provide an overview of the current treatment practice and outcomes of traumatic sternal fractures.

## Materials and methods

PubMed and EMBASE/Medline were searched with the terms ‘sternum’, ‘fracture’, ‘injury’, ‘treatment’, and their respective synonyms. Both searches were performed with a combination of free text entry terms and MeSH terms (PubMed) or Emtree terms (EMBASE/Medline). No filters or language restrictions were applied to the searches.

Primary and secondary outcomes for sternal fracture and dislocation treatment were defined (Table 1). Articles were eligible for inclusion if they were original studies on the treatment of traumatic sternal fractures and dislocations; had a human study population over 18 years of age; reported on > 1 primary outcome parameters; and had been published after 1990. Articles were excluded if they involved the treatment of non-traumatic sternal fractures or dislocations, or fractures caused by cardiopulmonary resuscitation, or if they were review articles. Due to the limited research performed on sternal injury, all types of original studies were included except case reports (i.e., articles with a study population of *n* = 1). All included articles were assessed for eligible cross-references. Finally, from all included articles, the parameters depicted in Table 1 were extracted.

**Table 1:** Parameters for the assessment of included articles

Study characteristics Year of publication Journal of publication Country Study type Study period Number of included patients Length of follow-up
Patient characteristics Age (mean and range) Gender (male or female) Type of sternal injury (fracture or dislocation) Location of sternal injury (manubrium, sternomanubrial joint, sternal body, xiphoid process) Associated injuries (isolated or combined sternal injury) Acute (< 1 month) or non-healing sternal fracture (> 3 months) (if applicable) Comorbidities
Treatment methods Type of treatment (surgical or conservative) Conservative treatment method (if applicable) Surgical indication (if applicable) Type of fixation material (if applicable)
Treatment outcomes Primary outcome parameters (Fracture) healing Sternal stability
Secondary outcome parameters Pain relief Treatment complications Removal of fixation material (if applicable) Other re-operation (if applicable) Hospital length of stay

The review of search results and the quality assessment were performed by two authors (DK and KW) independently. In case of disagreement, final consensus was reached through a thorough re-assessment of the relevant article.

Quality of included studies was assessed using the methodological index for non-randomized studies (MINORS) assessment criteria, a validated instrument for the assessment of comparative and non-comparative surgical studies [19]. In the current review, only the eight criteria for non-comparative studies were used. For each criterion, a score of 0, 1 or 2 points was awarded: 0 points were assigned if an item was not reported, 1 point if an item was reported but inadequate and 2 points if an item was reported and adequate, leading to a maximum of 16 points per study. An appropriate study endpoint was defined as confirmation of fracture healing or sternal stability, reported for all included patients. An appropriate follow-up period was defined as > three months follow-up.

Since many studies did not report outcome parameters for all patients, the number of evaluable patients varied for each outcome parameter. Hence, analyses were conducted with ratios and percentages. Treatment outcomes were evaluated in the general patient population and in subgroups of patients with different sternal injuries and treatment types. Due to the limited and incomplete data availability, no further subgroup analyses were conducted. Data were analysed using IBM SPSS Statistics, version 22.0 (Armonk, NY, USA).

## Results

### Search results

**Fig. 1:**
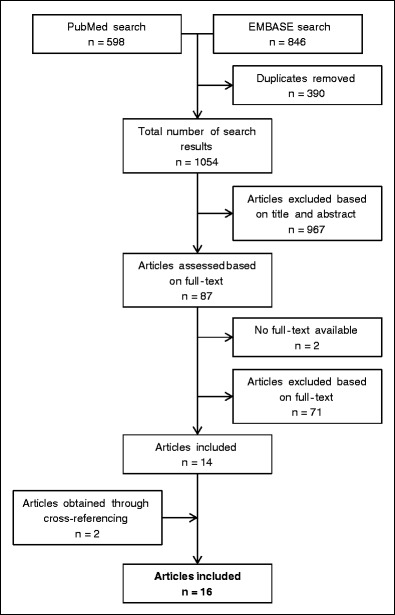
Search summary

The literature search was conducted on June 8, 2017. The PubMed and EMBASE/Medline searches generated 598 and 846 hits respectively, yielding a total of 1444 hits. After removal of 390 duplicates, the resulting 1054 articles were assessed based on title and abstract. Subsequently, 967 articles were excluded based on title and/or abstract showing no relevant data for the current analysis. The remaining 87 articles were assessed based on full-text and 14 of these articles were included. For two articles, a full-text version was not available and these articles were excluded. Additionally, through cross-referencing of the included articles, another two articles were obtained. A summary of the search process and search results is depicted in Fig. 1.

### Study characteristics

All 16 included studies were published between 2006 and 2017. There were 12 case series, two cross-sectional studies, and two prospective cohort studies. Study periods ranged from one to 13 years and follow-up length varied between one month and seven years. Although all studies together comprised 354 individual patients, many studies did not report primary outcome parameters for all patients. Therefore, only 191 patients were included in the analysis for this review (Table 2).

**Table 2:** Characteristics of included studies

Authors	Study type	Study period	*N*	Follow-up length
Abdul-Rahman et al. (2009) [23]	Case series	–	2 (primary outcomes available for *n* = 1)	8 weeks
Al-Qudah (2006) [24]	Case series	7 years	4	–
Ciriaco et al. (2009) [14]	Case series	6 years	6	2–7 years
Divisi and Crisci (2011) [7]	Cross-sectional study	16 months	11 (primary outcomes available for *n* = 8)	Mean 2 (1–3) months
Ergene et al. (2013) [25]	Case series	20 months	15 (primary outcomes available for *n* = 8)	–
Gloyer et al. (2011) [12]	Case series	–	3 (primary outcomes available for *n* = 2)	Mean 10 (6–12) months
Kälicke et al. (2006) [5]	Case series	–	2 (primary outcomes available for *n* = 1)	Mean 1.5 (1–2) years
Krinner et al. (2017) [2]	Case series	3 years	103 (primary outcomes available for *n* = 11)	2 years
Labbe et al. (2009) [13]	Case series	3 years and 5 months	11	–
Nazerali et al. (2014) [18]	Case series	7 years	57 (traumatic sternal fracture in *n* = 3)	3 months
Queitsch et al. (2011) [20]	Single-arm prospective cohort study	5 years	12	–
Richardson et al. (2007) [26]	Case series	13 years	35	–
Schulz-Drost et al. (2014) [27]	Prospective cohort study	1 year	10	6 months
Schulz-Drost et al. (2016) [8]	Cross-sectional study	22 months	13	12 weeks
Wu et al. (2005) [21]	Case series	1 year	6 (traumatic sternal fracture in *n* = 2)	6–18 months
Zhao et al. (2017) [1]	Case series	5 years	64 (primary outcomes available for *n* = 63)	6 months
Total	Case series (*n* = 12) Cross-sectional study (*n* = 2) Prospective cohort study (*n* = 1) Single-arm prospective cohort study (*n* = 1)	Mean: 52 months (range 1–13 years) Total: 56 years and 3 months	Total: *n* = 354 Included in analysis: *n* = 191	Range: 1 month–7 years

*N* number of patients, – not described

### Patient characteristics and treatment methods

**Fig. 2:**
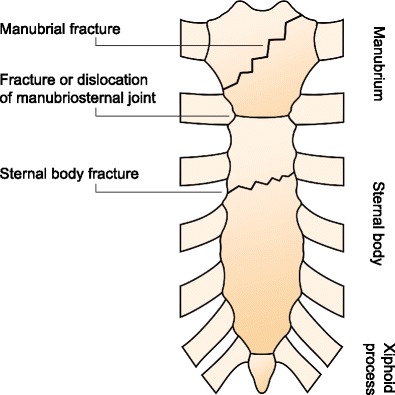
Anatomy of sternal injuries

Mean age was 38 years (range 17–88 years). There were 101 males (70%), 44 females, and 45 patients whose gender was not reported. Most patients (180/191, 94%) demonstrated a sternal fracture, most commonly located at the sternal body (30/64, 47%), followed by a fracture of the manubrium (16/64, 25%). Of these sternal fracture patients, 137 (77%) were treated for an acute fracture, while 42 (23%) suffered from non-union. Eleven patients (11%) displayed a sternal dislocation, all located at the manubriosternal joint. The anatomy of sternal injuries is depicted in Fig. 2.

The majority of patients (105/143, 73%) suffered from associated injuries. Frequently occurring associated injuries were rib fractures, haemothorax or pneumothorax, pulmonary contusion, spinal fractures, clavicular fractures, extremity fractures, and head injuries. However, associated injuries were not further analysed. Underlying comorbidities were not reported for any patient.

In total, 170 patients (89%) were surgically treated for their sternal injury. Of these patients, 141 (83%) underwent surgical fixation with plates, 28 (16%) with plates and bone graft, and one (1%) was treated with wires. The type of surgical plating varied per study: for instance, some studies used locking plates, while others used non-locking plates. Indication for surgery were fracture displacement or sternal dislocation, pain, respiratory insufficiency, sternal instability, sternal deformity, and fracture non-union. Most studies did not provide detailed information on the surgical indications. Hence, further analysis of surgical indications was not performed.

Twenty-one patients (11%) received conservative treatment. Only one study reported their conservative treatment method: passive reduction of the sternal fracture or dislocation by surgical fixation of the associated spinal fracture (Table 3).

**Table 3:** Patient characteristics and treatment methods

*N*	Mean age (range)	Gender	Type and location of sternal injury		Isolated or combined injury	Acute or non-healing fracture^a^	Surgical or conservative treatment	Fixation materials^b^
191	38 (17–88) years	Male 101/145 (70%)	Fracture 180/191 (94%)	Manubrium 16/64 (25%)	Isolated injury (38/143, 27%)	Acute fracture (137/179, 77%)	Surgical treatment 170/191 (89%)	Plates 141/170 (83%)
		Female 44/145 (30%)		Manubriosternal joint 12/64 (19%)	Combined injury (105/143, 73%)	Non-healing fracture (42/179, 23%)	Conservative treatment 21/191 (11%)	Wires 1/170 (1%)
				Manubriosternal joint and body 1/64 (2%)				Plates with bone graft 28/170 (16%)
				Manubrium and body 5/64 (8%)				
				Sternal body 30/64 (47%)				
				Xiphoid process 0/64 (0%)				
			Dislocation 11/191 (6%)	Manubriosternal joint 11/11 (100%)				

All ratios and percentages were calculated with the data available. Therefore, the number of patients analysed per parameter might not equal the total population number

*N* number of patients, – not described

^a^Acute or non-healing fracture: only applicable to sternal fractures

^b^Fixation materials: only applicable to surgical treatment

### Treatment outcomes

All patients in this review demonstrated sternal healing (187/187, 100%) and/or sternal stability (35/35, 100%) after either conservative or surgical treatment. In virtually all patients (133/136, 98%), treatment resulted in pain relief. Three patients (3/174, 2%), all treated surgically for an acute sternal fracture, suffered from treatment complications: one patient showed post-operative wound seroma, one patient was re-operated due to loosening of fixation materials, and one patient suffered from an intra-operative bleeding due to injury to the mammary artery (without further post-operative complications). In 15 cases (15/145, 10%), removal of osteosynthesis was reported: indications varied between patient discomfort and insurance reasons. However, several studies did not specify the indication for osteosynthesis removal. Mean length of hospital stay was 15 days (range 3 to 59 days), the length of stay was however often not reported (Table 4).

**Table 4:** Treatment outcomes

		*N*	Isolated injury	(Fracture) healing	Sternal stability	Pain relief	Treatment complications	Removal of fixation material^a^	Other re-operation^a^	Mean (range) hospital LOS in days
All patients		191	15/67 (22%)	187/187 (100%)	35/35 (100%)	133/136 (98%)	3/174 (2%)	15/145 (10%)	1/89 (1%)	15 (3–59)
Acute fracture	Surgical treatment	117	14/43 (33%)	113/113 (100%)	33/33 (100%)	98/101 (97%)	3/114 (3%)	3/73 (4%)	1/73 (1%)	15 (3–59)
	Conservative treatment	20	0/20 (0%)	20/20 (100%)	–	–	0/20 (0%)	N/a	N/a	–
Non-healing fracture	Surgical treatment	42	0/1 (0%)	42/42 (100%)	2/2 (100%)	32/32 (100%)	0/30 (0%)	1/14 (7%)	0/14 (0%)	12
	Conservative treatment	–	–	–	–	–	–	N/a	N/a	–
Sternal dislocation	Surgical treatment	10	1/2 (50%)	10/10 (100%)	–	2/2 (100%)	0/8 (0%)	2/10 (20%)	0/1 (0%)	5 (4–6)
	Conservative treatment	1	0/1 (0%)	1/1 (100%)	–	–	0/1 (0%)	N/a	N/a	–

All ratios and percentages were calculated with the data available. Therefore, the number of patients analysed per treatment group might not equal the total population number

*N* number of patients, *N/a* not applicable, – not described, *LOS* length of stay

^a^Removal of fixation material and other re-operation: only applicable to surgical treatment group

### Quality assessment

The mean total quality score of the included studies was 6.7 out of 16 (range 3 to 10). Most studies had appropriate endpoints to study aim (10/16) and a loss to follow-up below 5% (14/16). No study reported an unbiased assessment of study endpoints or a prospective calculation of sample size. Two studies reported their data collection methods, one of which collected data prospectively. Patient inclusion criteria were described in three studies, all of which included patients consecutively. Six studies clearly stated their study aim and nine studies had an appropriate follow-up period (Table 5).

**Table 5:** MINORS quality assessment

Study	Clearly stated aim	Inclusion of consecutive patients	Prospective collection of data	Endpoints appropriate to study aim	Unbiased assessment of study endpoint	Follow-up period appropriate to study aim	Loss to follow-up < 5%	Prospective calculation of study size	Total quality score
Abdul-Rahman et al. (2009) [23]	1	0	0	1	0	1	2	0	4
Al-Qudah (2006) [24]	1	2	0	2	0	0	2	0	7
Ciriaco et al. (2009) [14]	1	2	0	2	0	2	2	0	9
Divisi et al. (2011) [7]	2	0	0	1	0	1	0	0	4
Ergene et al. (2013) [25]	1	0	0	1	0	0	2	0	5
Gloyer et al. (2011) [12]	2	0	0	1	0	2	2	0	8
Kälicke et al. (2006) [5]	0	0	0	1	0	2	2	0	5
Krinner et al. (2017) [2]	1	0	0	1	0	2	2	0	7
Labbe et al. (2009) [13]	1	0	0	2	0	0	0	0	3
Nazerali et al. (2014) [18]	2	0	1	2	0	2	2	0	9
Queitsch et al. (2011) [20]	2	2	2	2	0	0	2	0	10
Richardson et al. (2007) [26]	1	0	0	2	0	1	2	0	6
Schulz-Drost et al. (2014) [27]	2	0	0	2	0	2	2	0	8
Schulz-Drost et al. (2016) [8]	1	0	0	2	0	2	2	0	7
Wu et al. (2004) [21]	1	0	0	2	0	2	2	0	7
Zhao et al. (2017) [1]	2	0	0	2	0	2	2	0	8
Mean quality score (range)	1.3 (0–2)	0.4 (0–2)	0.2 (0–2)	1.6 (1–2)	0 (0)	1.3 (0–2)	1.8 (0–2)	0 (0)	6.7 (3–10)

0 not reported, 1 reported but inadequate, 2 reported and adequate

## Discussion

Few studies have been conducted on the treatment outcomes of traumatic sternal fractures and dislocations and to date, no randomised controlled trials have been published. Most studies included in this review were case studies, with only two cross-sectional studies and two cohort studies available. Case studies lack a randomised or consecutive methodological approach and are thus prone to selection and publication bias. Since case studies typically report on remarkable patients and treatment outcomes, their results do not reflect the findings in a general patient population. Notably, in the current review, most studies were of poor quality, with a mean total quality score of 6.7 out of 16. For this reason, results of this review should be carefully interpreted.

In total, 16 studies with 191 patients were included in this review. The majority of patients suffered from associated injuries (73%) and underwent surgery (89%). All patients displayed sternal healing and/or sternal stability, with a complication rate of only 3%.

Due to the limited research available, standardised treatment guidelines for traumatic sternal fractures and dislocations are lacking. Most notably, information about conservative and surgical treatment indications and long-term treatment outcomes, both in terms of functional outcome and health-related quality of life, could significantly improve the treatment of these injuries.

In literature, one systematic review has been published, which reported on surgical treatment of sternal fractures [4]. The current review evaluated both surgical and conservative treatment, as well as treatment of sternal dislocations. Also, more studies were included in this review (16 compared to 12 studies in the review by Harston et al.) [4].

Sternal fractures and dislocations are rare injuries [1–3, 5], which was confirmed by the current review. The included studies comprised only 354 patients (of whom 191 patients could be analysed) in a total study period of 56 years and 3 months. Although only patients over 18 years of age were included in this review, one study [20] reported an age range of 17–54 years. Since the mean age of the patients was 33 years, we decided not to exclude this study from our analysis.

In accordance with literature [3, 4, 10], sternal injury mostly occurred in young male patients and most fractures were located at the sternal body. Since one of the included studies exclusively assessed manubrial fractures and did not report outcome data for patients with other sternal fractures [8], the incidence of manubrial fractures might be overestimated in our analysis.

In literature, the majority of sternal fractures occurs as isolated injuries and are treated conservatively [1, 3, 10, 14]. However, in this review, the majority of patients (89% of all patients and 85% of patients with an acute sternal fracture) received surgical treatment. Many included studies reported that some of their patients received conservative treatment, but did not include this conservative treatment group in the follow-up. Moreover, only 22% of patients in the current analysis sustained an isolated sternal injury. This overrepresentation of surgically treated and polytrauma patients could be explained by the lack of consecutive patient inclusion and complete follow-up in case series. Also, publication bias could have caused the underrepresentation of conservatively treated patients in literature.

Fracture non-union is a rare entity in sternal fractures, with an incidence of < 1% in literature [20, 21]. Nonetheless, 23% of our patient population was treated for fracture non-union. This difference could be explained by the fact that the majority of patients in this review was treated surgically, and sternal non-union is generally considered an indication for surgical treatment [4].

Not one study reported on underlying comorbidities in their patients. Hence, although this review focussed on the treatment of traumatic sternal fractures and dislocations, it was impossible to assess whether patients suffered from osteoporosis or other underlying bone diseases.

Almost all surgically treated patients underwent sternal fixation with plates (83%) or a combination of plates with bone graft (16%). Former studies have shown that sternal plating provides more stability and better chest wall function, as well as a decreased chance of non-union and improved bone healing, compared to wires [1, 2, 4, 17]. While Harston [4] found that 32% of all patients underwent surgical fixation with wires, it seems that surgeons have increasingly embraced the biomechanical advantages of plating. Bone graft is often used for the treatment of fracture non-union, due to its osteoinductive properties [7, 22]. Indeed, most patients receiving bone graft (70%) were treated for non-union, while in the other patients, bone graft was used for extra fusion between plate and bone after sternal dislocation.

In correspondence with the findings of Harston et al. [4], operative treatment of sternal fractures and dislocations seems to be safe and effective. All patients in this review displayed (fracture) healing and/or sternal stability. Only 3% of patients suffered from treatment complications and 1% needed re-operation. Harston et al. [4] found that 19% of surgically treated patients suffered from complications. This high percentage could be explained by the fact that osteosynthesis removal was defined as a complication. In the current review, authors of the included studies did not seem to consider removal of osteosynthesis a complication, since removal was reported separately from complications and reasons for removal were often not specified.

Only 21 patients were included in the conservative treatment group of this review. Of these patients, 11 were treated by passive reduction of their sternal injury and ten patients received unknown non-surgical treatment. Although all patients in the conservative treatment group reached fracture healing and none suffered from complications, treatment methods could not be compared. Furthermore, the group is too small to generalise the findings.

Although most studies provided information on the occurrence of complications in their patients, comprehensive definitions and numbers were often lacking. Similarly, pain relief was often not defined nor quantified. Only one study [1] reported an average decrease in Pain Severity Score (PSS) for their patient population, although the authors did not report whether pain relief was experienced by all patients individually. Hence, for the analysis of both complications and pain relief in this review, data might be biased or incomplete. Notably, length of follow-up ranged from 1 month to 7 years. Some complications, such as sternal non-union, appear later than others; therefore, in some studies, follow-up for complications might have been incomplete.

The mean length of hospital stay was 15 days, but ranged from 3 to 59 days. Only few studies reported the length of stay: most of these reported a mean hospital stay of three to 12 days, while one study [2] demonstrated a prolonged mean stay of 31 days. This difference could be caused by the fact that in the latter study, all patients suffered from associated injuries, while in the other studies, the majority of patients presented with an isolated sternal fracture. The difference in hospital length of stay could be explained by the association between associated injuries and length of hospital stay found in literature [3].

This systematic review has several limitations. Firstly, many studies did not report all primary and secondary outcome parameters. Therefore, for each outcome parameter, analysis could be performed on only a limited number of patients; consequently, results could be highly skewed by the outcomes of an individual study. Secondly, most studies included in this review were low-quality case series, with potential selection and publication bias. Finally, the positive treatment results found in this review could not be extrapolated to the general population of sternal injury patients. Most notably, merely 191 patients were included in this review, with only 21 patients treated conservatively and 11 patients suffering from sternal dislocation. Moreover, it was impossible to assess how many patients who initially received conservative treatment ultimately required surgery. Furthermore, indications for surgery could not be verified.

In conclusion, both surgical and conservative treatment of traumatic sternal fractures and dislocations seem to be safe and effective. All patients evaluated in this review displayed sternal healing, while reported complication rates were as low as 3%. However, very limited research has been performed on this topic and only 191 patients could be included in the current analysis. Available evidence consists mainly of case series with low scores on quality assessment. Consecutive cohort studies and randomised controlled trials are lacking and study results should be interpreted with caution. Both additional high-quality research and comprehensive information from patient registries are essential to verify surgical indications and treatment outcomes in the relevant patient populations.
